# A Novel miRNA Located in the *HER2* Gene Shows an Inhibitory Effect on Wnt Signaling and Cell Cycle Progression

**DOI:** 10.1155/2022/7216758

**Published:** 2022-06-14

**Authors:** Zahra Shabaninejad, Seyed Javad Mowla, Fatemeh Yousefi, Bahram Mohammad Soltani

**Affiliations:** Department of Molecular Genetics, Faculty of Biological Sciences, Tarbiat Modares University, Tehran, Iran

## Abstract

Human epidermal growth factor receptor 2 (HER2) is involved in the development of the majority of cancers. Therefore, it can be a potential target for cancer therapy. It was hypothesized that some of the broad effects of *HER2* could be mediated by miRNAs that are probably embedded inside this gene. Here, we predicted and then empirically substantiated the processing and expression of a novel miRNA named *HER2-miR1*, located in the *HER2* gene; transfection of a DNA fragment corresponding to *HER2-miR1* precursor sequence (*preHER2-miR1*) resulted in ~4000-fold elevation of *HER2-miR1* mature form in HEK293t cells. Also, the detection of *HER2-miR1* in 5637, NT2, and HeLa cell lines confirmed its endogenous production. Following the *HER2-miR1* overexpression, TOP/FOP flash assay and RT-qPCR results showed that Wnt signaling pathway was downregulated. Consistently, flow cytometry results revealed that overexpression of *HER2-miR1* in Wnt^+^ cell lines (SW480 and HCT116) was ended in G1 arrest, unlike in Wnt^−^ cells (HEK293t). Taking everything into account, our results report the discovery of a novel miRNA that is located within the *HER2* gene sequence and has a repressive impact on the Wnt signaling pathway.

## 1. Introduction

Human epidermal growth factor receptor 2 (*HER2*), frequently called HER2 receptor tyrosine kinase 2, is the most oncogenic member of the human epidermal growth factor (EGFR) family [1, 2]. Often, the HER2 receptor indirectly binds to its ligands and forms heterodimers with other *EGFR* receptors, preferentially with *ErbB3* [3, 4]. Dimers containing HER2 can activate a variety of signaling pathways such as MAPK, protein kinase C (PKC), and AKT pathways [5]. Based on the cellular context and HER2 partner, a wide range of cellular responses could be assigned by following HER2 activation, including proliferation, migration, adhesion, differentiation, and apoptosis. Furthermore, heterodimers prolong downstream signaling activation and increase their outputs in comparison with EGFR homodimer members [6, 7].

MicroRNAs (miRNAs) are a group of noncoding RNAs with ~ 22 nucleotides long that control numerous cellular process like survival, apoptosis, differentiation, and tumorigenesis. miRNAs can bind to the 3′-UTR segment of mRNAs and adjust the expression of their targets via either mRNA degradation or interference in protein translation. [8–11]. Lin4 and let7 as the first miRNAs were discovered in *C. elegans* using forward genetics methods [12]. However, due to the small sizes of miRNAs, and their susceptibility to mutations, the identification of novel miRNAs using forward genetics approaches is very difficult [13, 14]. On the other hand, several bioinformatics tools are developed to find human miRNA hairpin structures. These software programs are developed to find the main criteria of miRNAs including the stem-loop structure of miRNA precursors, phylogenic conservation, and thermodynamic stability of stem-loops as well as the genomic location of validated miRNAs [10, 14]. About 55000 *bona fide* miRNA genes have been guessed to be in the human genome, and ~2500 human mature microRNAs have been registered in the miRBase database (http://www.mirbase.org/) to date [15, 16]. Following our previous successful bioinformatics prediction and experimental validation of multiple novel miRNAs [3, 8, 17, 18], here, we intend to search the conserved region of *HER2* gene to find stem-loop structures, potentially encoding novel miRNAs. A potential hairpin structure located in the 5^th^ intron of *HER2* gene had all the features of the *bona fide* miRNA precursor, and the production of a mature miRNA was verified experimentally. Overexpression of the implied miRNA produced expected phenotypes, such as the alteration of *c-Myc*, *APC1*, and *APC2* gene expression and also suppression of the Wnt signaling pathway.

## 2. Materials and Methods

### 2.1. Bioinformatics Prediction

Using SSC profiler (http://mirna.imbb.forth.gr/SSCprofiler.html) [19], MiPRED (http://www.bioinf.seu.edu.cn/miRNA) [20], and Mireval (https://omictools.com/mireval-tool) [21] online tools, *HER2* gene was scanned to find possible stem-loop structures [19, 21]. miR-FIND (http://140.120.14.132:8080/MicroRNAProject-Web/), MaturePred (http://nclab.hit.edu.cn/maturepred/) [22], and MatureBayes (http://mirna.imbb.forth.gr/MatureBayes.html) [23] online tools were utilized to predict the Dicer and Drosha processing sites and also for prediction of probable mature miRNAs. UCSC database (http://genome.ucsc.edu/) was used to examine phylogenic conservation and also to find expression level of the region of interest available in deep sequencing data [24, 25]. Furthermore, the possible structure of the interested sequence was predicted by RNAfold software (http://rna.tbi.univie.ac.at/cgi-bin/RNAfold.cgi) [24]. DIANA MR-MicroT (http://diana.imis.athena-innovation.gr/DianaTools/index.php?r=mrmicrot/index) [26] was used along with RNAhybrid (http://bibiserv.techfak.uni-bielefeld.de) [27] to find the putative target genes of novel interested miRNA. DAVID (http://david.abcc.ncifcrf.gov/) tool was employed to determine the pathways in which novel miRNA might be involved. According to the Protein Atlas [28] (https://www.proteinatlas.org/) and EMBL-EBI (http://www.ebi.ac.uk/gxa/) databases, the *HER2* gene (and presumably *HER2-miR1*) is slightly expressed in HEK293t cell line.

### 2.2. Cell Lines

SW480, HT29, HCT116 (originated from colorectal carcinoma), A172 (glioblastoma), KYSE-30 (esophagus cancer), and 5637 (bladder carcinoma) were cultured in RPMI1640 media (Gibco), and HUH7, NT2, HEK293, and HeLa cell lines which are derived from hepatocellular carcinoma, malignant pluripotent embryonal carcinoma, embryonic kidney, and cervix carcinoma, respectively, were cultured in DMEM-HG (Gibco). These culture media were supplied with 10% fetal bovine serum (FBS) (Gibco) and with 100 *μ*g/ml streptomycin (Sigma) and 100 U/ml penicillin. The cells were incubated at 37°C and 5% CO_2_. The cell lines were purchased from Pasteur Institute, Iran, and National Center for Genetic and Biological Reserves in Iran.

### 2.3. Total RNA Extraction

Total RNA was extracted using Trizol (Invitrogen) according to the manufacturer's protocol. DNA contamination was degraded using RNase-free DNase I (Takara).

### 2.4. DNA Constructs

To clone the DNA of the predicted *preHER2-miR1*, a piece of the human *HER2* gene, about 300 bp, was PCR amplified using Int-5-F and Int-5-R primers (Table 1). This amplicon was cloned in the pEGFP-C1 expression vector (Clontech) downstream of the GFP gene. A sequence forming the hairpin structure that was cloned earlier into the pEGFP-C1 vector [8] was used as a scrambled negative control. All of these recombinant constructs were isolated and sequenced for confirmation of the right inserts.

### 2.5. Overexpression and Knockdown of *HER2-miR1* in Cell Lines

The studied cell lines which were cultured in 24 well plates were transfected with two micrograms of recombinant pEGFP-C1 vector containing HER2-miR1 precursor which were covered by Lipofectamine 2000 (Invitrogen). Mock and scrambled vectors were used as negative controls. After twenty-four hours of transfection, GFP expression was assessed with an invert fluorescence microscope (Nikon eclipse Te2000-s).

### 2.6. Analysis of the Cell Cycle

Cells were transfected with a *HER2-miR1* overexpression cassette and after 36 hours of transfection were harvested and dyed with propidium iodide (PI) (Roche). Triton X100 and RNase A were used to make the cells PI permeable and remove the cell's RNA, respectively. All assays were carried out using a FACS flow cytometer and analyzed with Cell Quest software (BD Biosciences).

### 2.7. Detection of *HER2-miR1*

RNA was extracted from HEK293t cells forty-eight hours after transfection with a *preHER2-miR1* overexpression cassette. Then, 3′-Poly-A tail was added to RNAs in a reaction containing 5 U Poly-A polymerase (Biolabs), 4 *μ*l 10 mMol ATP and 2 *μ*g of extracted RNA. According to our previously described protocol, the first-strand cDNA was synthesized using ReverseAid Reverse Transcriptase (Thermo Science) utilizing the specific anchored-oligo-dT primers [10]. Real-time quantitative PCR (RT-qPCR) was performed in an ABI PRISM 7500 system (Applied Biosystems), according to the following run method: the initial denaturation 15 min at 95°C and 48 cycles of 95°C/15 s, 65°C/20 s, and 75°C/15 s. RT-qPCR was performed according to MIQE guidelines using EvaGreen master mix (Amplicon). *GAPDH* and *U48* small nucleolar RNA (*SNORD48*) were used as reference genes [29, 30]. The RT-qPCR data were analyzed using the 2^-*Δ*CT^ and 2^-*ΔΔ*CT^ method [31].

### 2.8. TOP/FOPflash Assay

TOP/FOP reporter assays were performed with Dual-Glo Luciferase Kit based on the manufacturer's instructions (Promega). In brief, the SW480 cells were transiently cotransfected with TOP or FOPflash constructs (1 *μ*g) and *preHER2-miR1* construct and also scrambled and mock negative vector (1 *μ*g) in triplicate. TOPflash was assayed forty-eight hours after transfection for each vector.

### 2.9. Statistical Analysis

The GraphPad Prism 5.04 (GraphPad, San Diego, CA, USA) was used for the statistical analysis. Flow cytometry results (PI test) were interpreted with flowing software 2.5.1 (Flowing software, Turku, Finland). In all analyses, *P* value < 0.05 was considered statistically significant. Each experiment was performed in duplicate, and the assays were replicated at least two times. The data that support the findings of this study are available from the corresponding author upon reasonable request.

## 3. Result

### 3.1. Prediction of a Novel miRNA within the Intron of the Human *HER2* Gene

**Figure 1:**
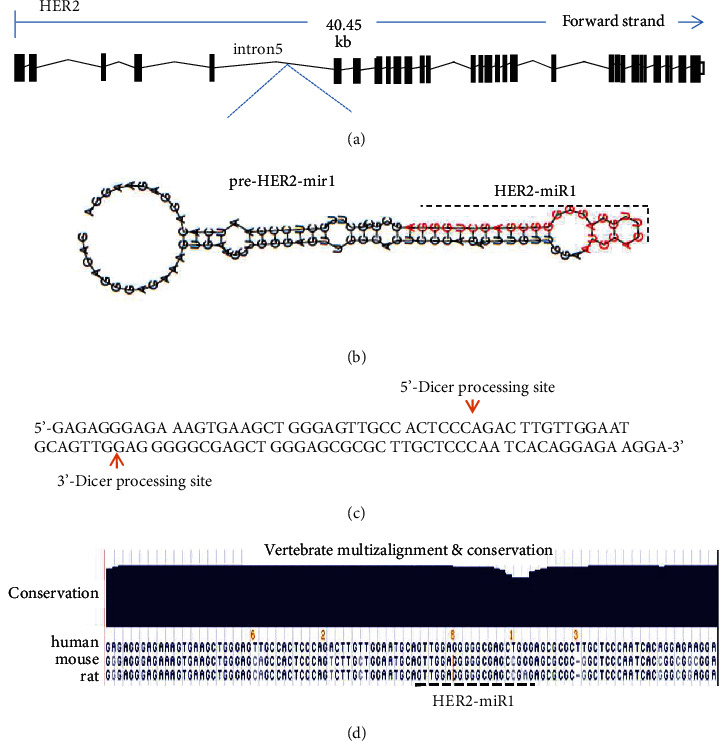
Bioinformatics prediction of *preHER2-mir1* within the 5^th^ intron of human *HER2* gene. (a) Schematic presentation of *HER2* gene adapted from Ensemble. Exons and introns are shown with a rectangular shape and broken lines, respectively. (b) Shows predicted stem-loop encoding *HER2-miR1*. The red-colored sequence is predicted by SSC profiler as possible *HER2-miR1* mature form. (c) Illustrates Drosha cutting sites on the sequence of *HER2-miR1* stem-loop predicted by miRFIND tool. (d) Blat search result by UCSC Genome Browser shows strong conservation of *HER2-miR1* among several organisms including mammals.

SSC profiler program was used to predict possible stem-loop structures within the HER2 gene (Figure 1(a)). This program demonstrated about 80 stem-loops within its exons and introns. One of these stem-loops, hg17, chr17: 35109679-35109728 that here we named pre-HER2-miR1 (Figure 1(b)), had most of the criteria for producing a real mature miRNA, named HER2-miR1. miR-FIND, mature Pred, and MatureBayes along with MiPRED recognized pre-HER2-miR1 as a real miRNA precursor with a significant score. Moreover, the UCSC Genome Browser blast search illustrated that the HER2-miR1 and its precursor are conserved in mammals (Figure 1(d)). Based on the Mireval online tool, HER2-miR1 precursor is strongly conserved and is not homologous with other validated miRNAs. Alignment of *HER2-miR1* to mature miRNAs which are registered showed only weak similarity to *hsa-miR-4687-3p* and *bra-miR-164e-3p*.

### 3.2. Detection of Exogenous *HER2-miR1* in HEK293t Cell Line

**Figure 2:**
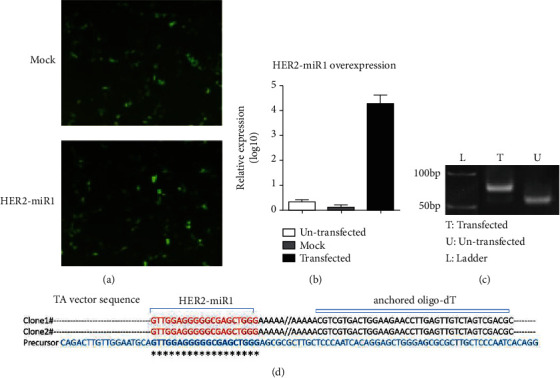
Production of exogenous *HER2-miR1* mature form from its precursor. (a) Fluorescent image (×40) of HEK293t cells transfected with mock vector or the expression vector that carries *HER2-miR1* precursor. (b) RT-qPCR results indicated the overexpression of *HER2-miR1* (~×4,000 folds) in the HEK293t cells that were transfected in (a). Data were normalized against *U48*, and error bars indicate standard deviation (SD) of duplicated experiments. (c) Electrophoresis gel image demonstrated the appropriate size of mature *HER2-miR1* in the cells overexpressing *preHER2-mir1* in comparison with un-transfected cells. (d) Sequencing result of two TA vector clones (named clones 1 and 2) containing *HER2-miR1*, which is aligned with *preHER2-mir1*. 3′-end of *HER2-miR1* was at least three nucleotides longer than the specific primer shown by a black arrow which was used for amplification of this miRNA. The downstream and upstream nucleotides to *HER2-miR1* sequence belong to the vector and anchored oligo-dT primer.

In an attempt to detect mature *HER2-miR1*, the *preHER2-miR1* construct was overexpressed in HEK293t cell line. The pEGFP-C1 empty vector (mock vector) and untransfected cells were used as negative controls. The efficiency of transfection was estimated based on visual observation of GFP emission, and the best transfected cells were then selected for RNA extraction (Figure 2(a)). The RT-qPCR analysis showed that for the cells overexpressing *preHER2-miR1*, the *HER2-miR1* expression level was increased about 4,000 folds compared to the cells in which the mock vector was overexpressed (Figure 2(b)). Gel electrophoresis proved the right size for the amplification products of exogenous *HER2-miR1* by RT-qPCR (Figure 2(c)). When RT-qPCR products with the expected size (about 80 bps) were sequenced (Figure 2(d)), the result revealed the efficient production of mature *HER2-miR1*. The minimum size of this sequence was submitted to EMBL-EBI database under the accession number # PRJEB10344.

### 3.3. Detection of Endogenous *HER2-miR1* in Various Human Cell Lines

**Figure 3:**
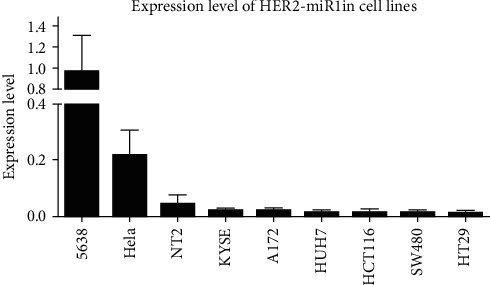
Detection of *HER2-miR1* in various cell lines. The maximum expression level of *HER2-miR1* was detected in 5637 cells, and the minimum was detected in colorectal cancer-originated cell lines, SW480 and HT29. *U48* RNA was used for normalizing the expression levels of *HER2-miR1*. Error bars reveal the SD of duplicated experiments.

To examine whether the *HER2-miR1* is expressed endogenously, RNA samples were extracted from various cell types, including 5637, Hela, NT2, KYSE, A172, HUH7, SW480, HT29, and HCT116 cell lines. Then, *HER2-miR1* was especially amplified in the cDNAs prepared from the mentioned RNA extracts, using RT-qPCR. Our results showed that the 5637 cells had the highest level of *HER2-miR1*. On the other hand, the NT2 and HeLa cell lines had a moderate level of *HER2-miR1*, while no significant expression was observed for this novel miRNA in other tested cell lines (Figure 3).

### 3.4. Analysis of *HER2-miR1* Direct Interactions with Predicted Target Genes

**Figure 4:**
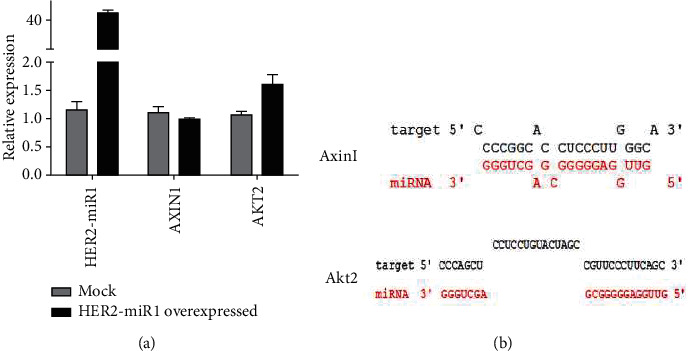
Direct interaction of *HER2-miR1* with its predicted target genes. (a) RT-qPCR indicated that HER2-miR1 alteration expression had significant effect on Axin1 and but not Akt2 genes at the RNA level in SW480 and HEK293T cells. (b) Shows the pairing status of *HER2-miR1* with predicted MREs in the 3′-UTR sequences of predicted target genes.

RNAhybrid tool predicted *Axin1* and *Akt2* genes as putative direct targets for *HER2-miR1* showing one MRE for each gene (Figure 4). Then, following the overexpression of HER2-miR1 and Anti-HER2-miR1, RT-qPCR data confirmed its elevated expression level up to ~30 and ~25 folds, respectively, 48 h after transfection of SW480 cell line. Also, data showed an elevate expression of HER2-miR1 and anti-HER2-miR1 cassette up to ~40 and ~30 folds, respectively, 48 h after transfection of HEK293T cell line. The intrinsic expression of HER2-miR1 was reduced by ~half, after transfection with anti-HER2-miR1 cassette in both cell lines (Figure 4(a)). RT-qPCR against Axin1 predicted target genes indicated significant expression alteration of this gene at the RNA level in SW480 and HEK293T cells. In overexpressed HER2-miR1 cells, the Axin1 expression showed a significant decrease (~0.5 and ~0.25 fold) in both cell lines in compassion with overexpressed mock cells. Also, in both cell lines, knowing down of HER2-miR1 by anti-HER2-miR1 cassette caused an about twofold increase in Axin1 expression (Figure 4(a)). But, the AKT2 expression, as one of predicted targets, showed no significant alternation.

### 3.5. *HER2-miR1* Overexpression Effect on the Wnt Signaling Pathway

**Figure 5:**
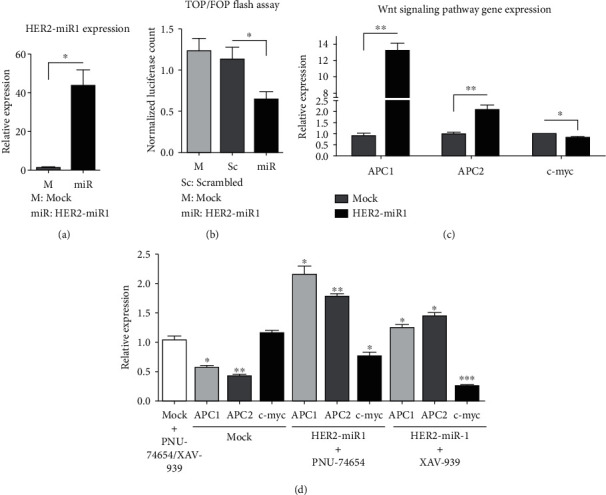
Involvement of *HER2-miR1* in Wnt signaling pathway regulation. (a) Accumulation of *HER2-miR1* in SW480 cells overexpressing *preHER2-miR1*. (b) Shows significant Wnt signaling downregulation following *HER2-miR1* overexpression. Relative luciferase activity was measured in SW480 cells which were cotransfected with TOP-flash vector along with *preHER2-miR1* overexpressing vector or mock and scrambled control vectors. Error bars indicate SD of triplicated experiments, *P* < 0.05. (c) Shows the upregulation of the *APC1* and *APC2* genes and downregulation of c-Myc, following *HER2-miR1* overexpression in SW480 cell line. Error bars indicate SD of duplicated experiments, *P* < 0.05. (d) Shows *HER2-miR1* overexpression along with PNU-4654 and XAV-939 small molecules effects on *APC1*, *APC2*, and *c-Myc* gene expression. Error bars indicate SD of duplicated experiments, *P* < 0.05.

To analyze the influence of *HER2-miR1* on the Wnt signaling pathway, the pGL4-TOP and *HER2-miR1* overexpressing vectors were transiently cotransfected into the SW480 cell line (Wnt^+^). In the control experiment, the *HER2-miR1* overexpressing vector was replaced with the mock or scrambled vectors. The elevated level of mature *HER2-miR1* in the SW480 cells transfected with the overexpression cassette of *HER2-miR1* was confirmed using RT-qPCR (Figure 5(a)).

To further investigate the influence of *HER2-miR1* overexpression on this pathway, three downstream genes were also measured with RT-qPCR in SW480 cells. The *APC1* and *APC2* gene expression levels were significantly upregulated after *HER2-miR1* overexpression in these cells (Figure 5(c)). However, the expression level of the *c-Myc* gene was significantly decreased in the cells overexpressing *HER2-miR1* (Figure 5(c)). To confirm this result, we used two small molecules, PNU-74654 and XAV-939, that inhibit the Wnt signaling pathway [17, 32]. SW480 cells were treated with these small molecules for 10 hours and then transfected with the *pre-HER2-miR1* construct. In both cases, the overexpression of *HER2-miR1* significantly upregulated APC1 and APC2 genes, whereas *c-Myc* expression level showed a reduction in the presence of these small molecules (Figure 5(d)).

### 3.6. Cell Cycle Analysis

**Figure 6:**
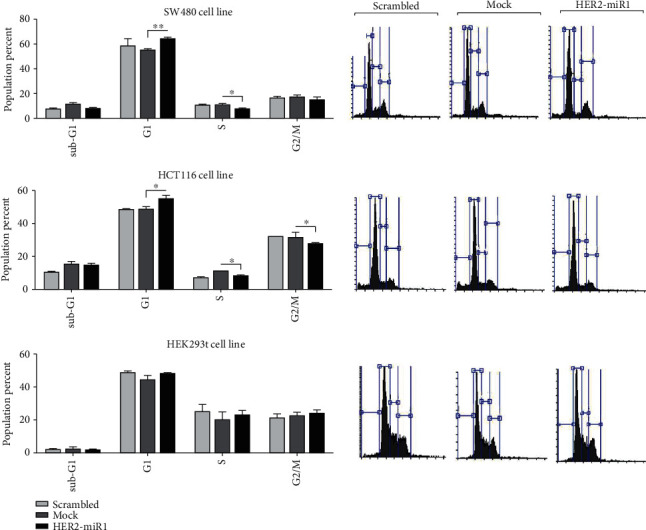
*HER2-miR1* overexpression effect on cell cycle status of Wnt^+^ and Wnt^−^ cells. (a, b) Show *HER2-miR1* overexpression effect on SW480 (a) and HCT116 (b) cell cycle distribution, 36 h after transfection, measured by PI. Overexpression of *HER2-miR1* resulted in significant G1 elevation and S phase reduction in both SW480 and HCT116 cell lines, compared to the related controls. (c) Overexpression of *HER2-miR1* did not significantly change the cell cycle population distribution of HEK293t cell line. Error bars indicate SD of duplicated experiments, *P* < 0.05.

SW480 and HCT116 (Wnt^+^) and HEK293t (Wnt^−^) cell lines were transfected with the vector overexpressing *HER2-miR1*. Then, the transfected cells were stained with PI and analyzed with the flow cytometer, 36 h after transfection. About 15% elevation of G1 and ~3.3% reduction of S phases were observed in SW480 cells following the *HER2-miR1* overexpression compared with the related controls (Figure 6(a)). Similarly, about 5% elevation of G1 and 4% reduction of S phases were perceived in HCT116 cells overexpressing *HER2-miR1* (Figure 6(b)). However, the overexpression of *HER2-miR1* did not alter the cell cycle of the HEK293t cell line compared to mock control, significantly (Figure 6(c)).

## 4. Discussion

MicroRNAs are key regulators of many biological processes including proliferation, differentiation, and apoptosis. Much attention is devoted to the miRNA detection, biogenesis, and function over the last decade, with the goal of novel pharmacological therapy [33]. Methods that rely on computational tools have accelerated the prediction of new miRNAs and their target genes [34]. miRNAs have a small size, have low expression level in tissues and cells, and show time-dependent expression. Therefore, forward genetics has been inefficient in miRNA gene detection [35]. Today, there are several miRNA prediction software programs that accelerate novel miRNA detection [34, 36].

SSC profiler predicted more than 80 stem-loop structures in the human *HER2* gene. One of these stem-loop structures that we called it *HER2-miR1* and located in its 5th intron (Figure 1(a)) had the most features for producing a *bona fide* miRNA (Figure 1(b)). Moreover, the miRFIND online tool prognosticated a Drosha processing site in this sequence (Figure 1(c)). Similar to most identified miRNAs [8, 10, 11, 37, 38], UCSC tool illustrated a high conservation pattern for *preHER2-miR1* and its mature form in several organisms including mammalians (Figure 1(d)). When high-score target genes, predicted by DIANA MR-MicroT, were categorized by DAVID online tool, it was suggested that *HER2-miR1* might regulate the Wnt signaling pathway. In addition, RNAhybrid predicted strong and poor complementation for *Axin1* and *Akt2* as the target genes for *HER2-miR1*, respectively (Figure 4(b)). Generally, all of these employed bioinformatics software programs strongly supported the presence of this novel miRNA in the *HER2* gene.

Protein Atlas [28] and EMBL-EBI databases indicated that the *HER2* gene (and probably *HER2-miR1*) is slightly expressed in HEK293t cell line, which is efficiently transfected [39]. Then, *preHER2-miR1* was overexpressed in these cells, and exogenous mature *HER2-miR1* was detected using the reported approach [8, 10] (Figure 2(a)). RT-qPCR amplification products of *HER2-miR1* overexpression (Figure 2(b)) with the expected size (Figure 2(c)) were cloned and sequenced. Two colonies had the same nucleotide sequences and well-matched to *preHER2-miR1*. MicroRNAs are described to be 18-27 nucleotides long [8, 10, 11, 40, 41]; here, *HER2-miR1* was at least 20 nucleotides long (Figure 2(d)).

Endogenous detection of a predicted novel miRNA is supportive evidence for its identity [14]. The cell lines originated from colorectal cancer, including HCT116, HT29, and SW480, presented the lowest level of *Her2-miR1* expression. It is consistent with the very low expression level of the *HER2* gene in SW480 that is reported elsewhere [42]. On the other hand, the bladder-originated 5637 cells, which express *HER2*, showed the highest expression level of *HER2-miR1* (Figure 3) [43]. Relatively higher expression of *HER2-miR1* in 5637 cell line suggests that this miRNA may be useful for further analysis in bladder cancer specimens as a diagnostic biomarker.

Hence, *HER2-miR1* was successfully overexpressed in SW480 (as a Wnt^+^) cell line (Figure 5(a)), and the Wnt pathway activity was evaluated using TOP/FOPflash assay system (Figure 5(b)). Our results showed that *HER2-miR1* downregulated the Wnt pathway compared with scrambled and mock controls (Figure 5(b)). Consistent with the downregulation of the Wnt signaling pathway (Figure 5(b)) and downregulation of *c-Myc* (Figure 5(c)) as a known downstream gene for the Wnt signaling (51), *APC1* and *APC2* genes as Wnt signaling inhibitors [44, 45] were upregulated, following the *HER2-miR1* overexpression (Figure 5(c)). Also, *HER2-miR1* showed synergic inhibitory effects on the Wnt signaling with XAV-939 and PNU-74654 small molecules (Figure 5(d)). Overall, these results suggest that *HER2-miR1* may play a role as a negative regulator of the Wnt signaling pathway.

Consistently, overexpression of *HER2-miR1* in both SW480 and HT29 as Wnt^+^ cells [46] resulted in the increased and decreased proportion of the cells at the G1 and S phase in transfected cells, respectively (Figures 6(a) and 6(b)). Such an effect was not detected in Wnt^−^ HEK293t cells (Figure 6(c)) [47]. These controversial cell cycle effects of *HER2-miR1* could be attributed to differences between physiological and cellular conditions and genetics and epigenetics backgrounds of the studied cell lines. In conclusion, here, we have presented *HER2-miR1* as a novel conserved miRNA mapped within the 5^th^ intron of the human *HER2* gene and provided pieces of evidence concerning its specifications and functionality in the adjustment of the Wnt signaling pathway, especially by upregulating APC1 and *APC2* gene expression. Considering the role of HER2 signaling in breast cancers, it has remained to be tested if *HER2-miR1* affects breast cancer initiation and progression.

## Figures and Tables

**Table 1:** Name and sequence of primers used in this research.

Primer name	Primer sequence 5′ to 3′
*HER2-miR1*	GTTGGAGGGGGCGAGCT
*U48*	TGACCCCAGGTAACTCTGAGTGTGT
Anchored oligo-dT	GCGTCGACTAGTACAACTCAAGGTTCTTCCAGTCACGACG(T)18 N
Universal outer	AACTCAAGGTTCTTCCAGTCACG
Universal inner	GCGTCGACTAGTACAACTCAAG
*GAPDH*	Forward: GTGAACCATGAGAAGTATGA Reverse: CATGAGTCCTTCCACGATAC
*APC1*-real time	Forward: TATTACGGAATGTGTCCAGCTTG Reverse: CCACATGCATTACTGACTATTGTC
*APC2*-real time	Forward: CGCACCCGTGAGGACTACAGGC Reverse: GATCATCTTGTGCTTGGAGTGCACC
*c-myc*-real time	Forward: CTCCTACGTTGCGGTCACAC Reverse: CGGGTCGCAGATGAAACTCT
*Axin1*-real time	Forward: ATGCAGGAGAGCGTGCAGGTC Reverse: TGACGATGGATCGCCGTCCTC
*Axin*1-3′UTR	Forward: AAGGTGGACTGATAGGCTGGT Reverse: AGAAGACACACCACAGCCAGG
*Akt2*-3′UTR	Forward: CAGCCTCCAGCCTCACCTTTG Reverse: TGTGCCCACACTACGAGACC

## Data Availability

No data were used to support this study.
